# An analysis of interventional radiology training needs of radiology registrars in South Africa

**DOI:** 10.4102/sajr.v29i1.3192

**Published:** 2025-10-10

**Authors:** Salman Idrees, Nicholas Christofides, Halvani Moodley

**Affiliations:** 1 Department of Radiology, School of Health Sciences, University of the Witwatersrand, Johannesburg, South Africa

**Keywords:** interventional radiology training, medical education in Africa, subspeciality training, South Africa, interest and exposure in interventional radiology, radiology subspecialisation

## Abstract

**Background:**

Interventional radiology (IR) is rapidly growing as a critical subspeciality, yet in South Africa (SA) IR training remains underdeveloped, with no national data to guide structured training and workforce expansion.

**Objectives:**

This study aimed to assess South African radiology registrars’ interest in IR, their exposure, and the existing training environment.

**Method:**

A cross-sectional, electronic quantitative and qualitative survey was conducted from 09 February 2024 to 08 March 2024 among registrars affiliated with the Radiological Society of South Africa. The survey gauged their interest in IR, exposure, training infrastructure, learning tools, satisfaction with training and potential areas for improvement.

**Results:**

Response rate: 45% (100/221 registrars); 54% were female, median age was 33.4 years and 51% in their first or second year. Interest in IR as a subspeciality was expressed by 40%, 27% were not interested and 33% were unsure. Only 27% reported adequate exposure; 56% had limited exposure and 17% had none. Dissatisfaction with IR training was noticed by 47%, 37% were undecided and 16% were satisfied. Only 9% had a structured curriculum, while 23% relied on mentorship. Major themes for improvement included structured training, mentorship, supervision, training facilities and hands-on exposure.

**Conclusion:**

Despite strong interest, radiology registrars (including international supernumerary registrars) face significant gaps in IR training and exposure in SA.

**Contribution:**

This is the first national study to provide essential baseline data to inform targeted reforms and development of structured, locally relevant IR training.

## Introduction

Interventional radiology (IR) is a rapidly growing medical subspeciality.^1,2^ Its global growth has been driven by the growing demand for minimally invasive procedures associated with reduced morbidity, shorter hospital stays, and lower costs compared to traditional surgical methods.^1,2^ In South Africa (SA), where public healthcare faces significant resource constraints,^3^ the cost-effectiveness and clinical value of IR^2^ make it especially relevant. In response to this growing demand, many countries have formalised IR training through dedicated curricula, fellowships and board certification pathways.^4,5^

Despite these advances, the availability of trained interventional radiologists in Africa remains limited.^6^ For example, Kenya has only 10 IR specialists for a population of 54 million^6^ and in Tanzania, efforts have been made to address this gap through the introduction of a state-certified IR fellowship programme.^7^ However, SA has not yet established a nationally recognised IR subspeciality certification. Training remains largely embedded within diagnostic radiology programmes at registrar level. Although isolated initiatives such as local fellowships are emerging,^8^ these are not yet widely accessible.

South Africa is facing a significant ‘brain drain’ with over 23 000 health professionals currently working in countries such as the United Kingdom (UK), United States, Australia and New Zealand.^9^ Limited training opportunities are one of the contributors to this outflow of skilled doctors.^9^ A 2012 survey in SA revealed that 18.4% of radiologists were interested in IR, with younger professionals (registrars and early-career consultants) expressing the greatest interest in pursuing IR training abroad.^10^ Without accessible and structured local IR training, the country risks losing even more young radiologists to international subspecialisation pathways.

Radiology registrarship is likely the first point of exposure to IR, and most IR training is initiated at registrar level. Early exposure, access to structured training, and mentorship are key factors that have been shown to increase interest and uptake among trainees.^5,11^ To date, no national-level study has been performed to gauge radiology registrar interest, exposure to IR, and satisfaction with IR training across SA. Therefore, expanding the IR workforce in SA depends heavily on improving these elements at the registrar level.

The objectives of this study were to assess registrars’ interest in pursuing a career in IR, evaluate their procedural exposure to IR during training, and examine the structure and perceived quality of current IR training environments.

## Research methods and design

This was a cross-sectional, electronic survey-based study using both quantitative and qualitative methods. The survey was developed after an extensive literature review. Numerous published surveys investigating similar objectives were used as a guide to help design and modify the survey.^1,11^ SurveyMonkey, an online tool, was used to create and administer the survey (Online Appendix 1).

The survey link emailed to participants was open for 1 month (09 February 2024 – 08 March 2024), and a reminder email was sent halfway through the survey period. The sample consisted of all registrars (221) affiliated with 9 South African universities. The survey contained 28 questions, 17 multiple choice questions and some which used a Likert scale. Four questions required numerical answers, and 5 questions had additional space for further responses. Qualitative data were collected with two open-ended questions. The survey gauged interest in IR, exposure, training infrastructure, learning tools, satisfaction with training and potential areas for improvement. Participants were asked to report their procedural exposure as per their College of Medicine of South Africa (CMSA) logbooks.

### Ethical considerations

Approval was obtained from the University of the Witwatersrand Human Research Ethics Committee (Medical)(reference number: M231023). The Radiological Society of South Africa (RSSA) granted approval to distribute the survey link to its members. A link to the electronic survey together with a consent sheet was emailed to the radiology registrars and supernumerary registrars on the RSSA database. The data were anonymised, secured under password protection in SurveyMonkey, and exported to Microsoft Excel. All data were stored on a password protected computer with access limited to the study authors (principal investigator, the supervisor, and the co-supervisor).

#### Data analysis and statistics

**Quantitative analysis:** The data were imported from Microsoft Excel (Microsoft, Redmond, WA, United States) worksheet into IBM SPSS version 28 (IBM Corp, Armonk, NY, United States) for statistical analysis. The survey response rate was computed as the percentage of the registrars who completed the survey. Descriptive statistics were used to present the study participants’ demographics as well as their responses to the survey questions. Categorical variables were reported using frequency and percentages. Continuous variables were reported using means and standard deviations when their distributions were approximately normal, or medians with interquartile ranges if the data were skewed. Normality of distribution of continuous data was examined using the Shapiro–Wilk test. The comparison of categorical results by the registrars’ demographic profiles was performed using the Fisher’s exact test and the Pearson Chi-square test. For comparisons on numeric variables, the independent samples *t*-test and ANOVA or the Mann–Whitney *U* test and Kruskal–Wallis test were used, depending on the normality of distribution of the data.

**Qualitative analysis:** Thematic analysis was used for analysing responses to open-ended questions, conducted within a critical realist framework. This approach recognises meaning and experience as subjective while also shaped by broader social and institutional contexts.^12^ It was chosen to explore how radiology registrars in SA experience IR training, focusing on challenges faced as well as areas of improvements.

Data were analysed using the six-phase method of thematic analysis described by Braun and Clarke^13^:

1. Familiarisation: The main author read through all responses to open-ended questions multiple times to become fully immersed in the data. Initial observations and ideas were noticed.
2. Generating initial codes: The principal investigator systematically coded the data using both deductive codes – aligned with the study’s research questions (e.g. IR interest, exposure, and training) – and inductive codes that emerged directly from the data. Because the questions focused on IR training, interest in IR wasn’t mentioned and so wasn’t coded.
3. Constructing themes: Related codes were grouped into subthemes and then organised into broader themes that reflected key patterns in the participants’ experiences and perceptions.
4. Reviewing themes: The themes were reviewed and refined to ensure internal coherence and meaningful distinctions between themes.
5. Defining and naming themes: Each theme was clearly defined, and representative quotes were identified to illustrate the key ideas and lived experiences conveyed by participants.
6. Producing the report: Thematic findings were integrated into the results section, with selected participant quotes used to support the narrative and ensure transparency. The major theme ‘exposure & hands-on experience’ recurred in response to the second open-ended question. To avoid repetition, recurring subthemes were removed.

To enhance trustworthiness, two co-authors reviewed the final themes and provided feedback to ensure they were conceptually sound and consistent with the dataset. In addition, Just Done AI, an artificial intelligence tool, was used to assist with theme validation.^14^ Artificial intelligence-generated suggestions were cross-verified against the manual analysis and incorporated only when consistent with the identified patterns.

This process enabled a rich, contextualised understanding of the training challenges registrars face and the improvements they value most in IR training.

## Results

### Demographics

A response rate of 45% (100/221) was achieved. Table 1 and Table 2 show the demographic characteristics and the university of the participants. Of the participants, 11% were identified as supernumerary registrars from Botswana, Namibia, Somalia and Zimbabwe.

**TABLE 1:** Demographics of survey participants (*N* = 100).

Variable	Category	%	Mean	s.d.
Age (years)		-	33.4	3.6
Gender	Male	46	-	-
	Female	54	-	-
Duration of registrar programme	4 years	66	-	-
	5 years	34	-	-
Year of study	1st	28	-	-
	2nd	23	-	-
	3rd	21	-	-
	4th	20	-	-
	5th	8	-	-

s.d., standard deviation.

**TABLE 2:** University of survey participants.

University	Total number of registrars	Participants	
		*n*	%
Sefako Makgatho Health Sciences University	17	4	23.5
Stellenbosch University	27	9	33.3
University of Cape Town	25	8	32.0
University of Free State	8	7	87.5
University of KwaZulu-Natal	15	7	46.7
University of Limpopo	15	3	20.0
University of Pretoria	25	5	20.0
University of the Witwatersrand	69	56	81.0
Walter Sisulu University	20	1	5.0

**Total**	**221**	**100**	**45.0†**

† , Response rate.

### Interest in interventional radiology

**FIGURE 1:**
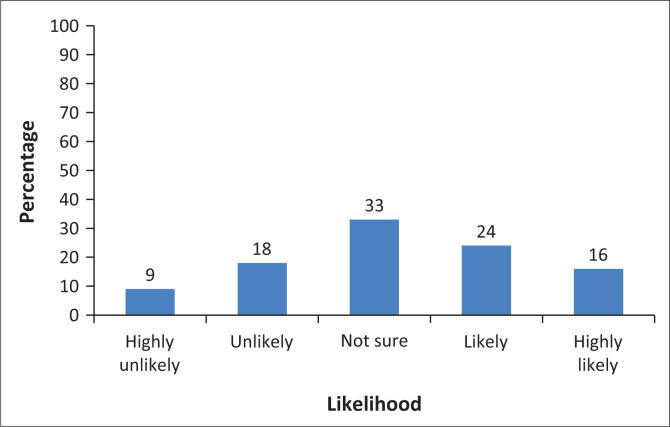
How likely are you to consider interventional radiology as a future sub-speciality? (*N* = 100).

Forty per cent of the participants would consider IR as a potential subspeciality (responded as highly likely and likely to be interested) (Figure 1). From participants who were either likely or highly likely to consider IR as a subspeciality, the most important factors for considering IR were financial factors (*p* = 0.01), not having another radiology subspeciality interest (*p* = 0.018), and the level of exposure to IR during registrar training (*p* = 0.049). The factors that did not show any significance included hours of work (*p* = 0.616), procedural-based work (*p* = 0.616), level of stress (*p* = 0.214), patient interaction (*p* = 0.538) and radiation exposure (*p* = 0.211). There was no correlation between the level of interest and gender (*p* = 0.186).

Among those who would not consider a subspeciality in IR (73%) (responded as not sure, unlikely, highly unlikely), there was still a desire to acquire the skill to perform various IR procedures. These procedures included ultrasound-guided biopsy (53%), abscess drainage (45%), percutaneous nephrostomy (PCN) insertion (42%), lung nodule biopsy (40%) and diagnostic angiograms (30%). A total of 1% of the participants also expressed an interest in learning additional procedures such as port insertion, peripherally inserted central catheter (PICC) line insertion, percutaneous biliary drainage, thyroid fine needle aspiration, arthrograms, joint aspiration, facet injections, perineural injections, and arterial embolisation procedures.

### Exposure to interventional radiology

**FIGURE 2:**
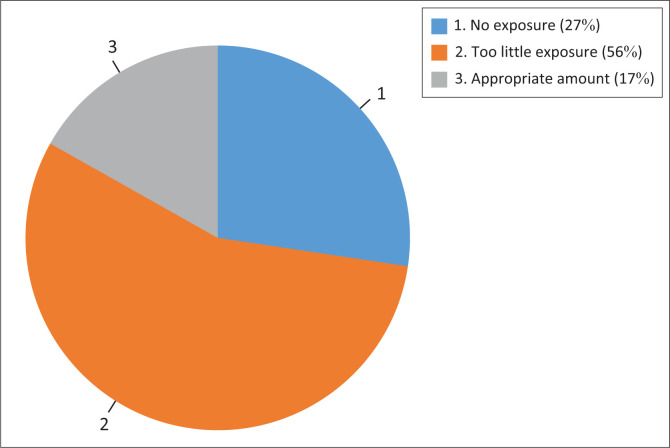
How much interventional radiology exposure have you had during your training so far? (*N* = 95).

**FIGURE 3:**
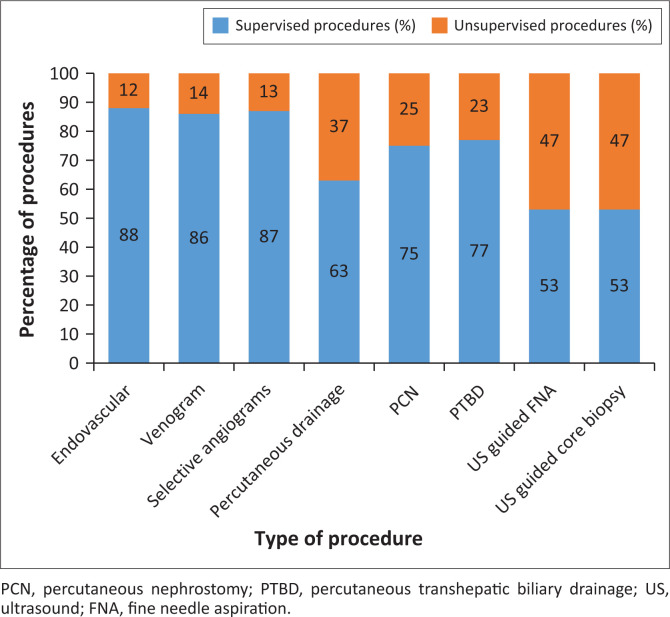
Percentage of procedures performed by registrars (supervised vs. unsupervised).

Only a minority of participants (17%) reported having adequate exposure (Figure 2). A wide variety of procedures are performed during rotations (Figure 3). As the year of study increases, there is a higher likelihood that registrars report having too little exposure (*p* < 0.001). No statistically significant differences exist between male and female participants and their reported level of exposure (*p* = 0.067). The mean number of weeks spent in an IR rotation was 6.7 weeks (s.d. = 3.7 weeks), ranging from 1.1 weeks to 10.7 weeks.

Participants from two of the nine universities reported not having any exposure to IR. One of these universities only had one participant and the other had three. However, all of these registrars were in their final year of training.

For all supervised procedures (except venograms), the number of procedures performed increased with the year of study (*p* < 0.001). The number of supervised venograms performed also increased with the year of study (*p* < 0.005). However, there was no correlation between the year of study and unsupervised angiographic procedures performed (interventional angiographic procedures, venograms and selective organ and/or subregional angiograms). Unsupervised, percutaneous procedures increased with the year of study (percutaneous draining procedures: *p* = 0.001; PCN: *p* = 0.002; percutaneous transhepatic biliary drainage *p* = 0.004; ultrasound-guided fine needle aspiration [FNA] *p* < 0.001; ultrasound-guided core biopsy *p* < 0.001). No statistically significant correlation was found between males and females on either supervised or unsupervised procedures (all *p* > 0.05; *p*-values ranged from 0.117 to 0.899).

Interventional radiology on-call responsibilities were reported by a minority (25%) of participants. On-call duties encompassed procedures such as abscess drainage (88%), PCN (63%), peripheral or other types of angiograms (21%) and cerebral angiograms (17%). A significant association existed between lower satisfaction with IR training and both insufficient exposure (*p* < 0.001) and absence of on-call duties (*p* < 0.001).

Supernumerary registrars were more likely to report having had either too little exposure or no exposure (*p* = 0.03). Sixty-three per cent of supernumerary registrars reported not having had their 1st hands-on exposure to IR, and all of these registrars were either in their first or second year of registrar training at the time of the survey.

### Interventional radiology training

**FIGURE 4:**
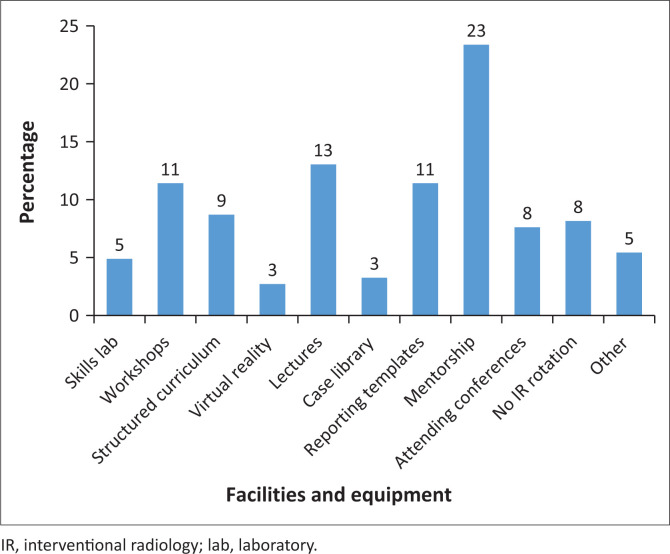
Facilities or equipment used for training.

A wide range of tools are being used for IR training across the country (Figure 4). There was a trend towards an association between having IR lectures and registrars’ interest in the field (*p* = 0.051), but this did not reach statistical significance. Access to the remaining tools and facilities showed no statistical correlation with registrar’s interest in IR (all *p* > 0.05; *p*-values ranged from 0.195 to 0.963). The ‘Other’ category represented open-ended answers such as ‘See one, do one, teach one’.

**FIGURE 5:**
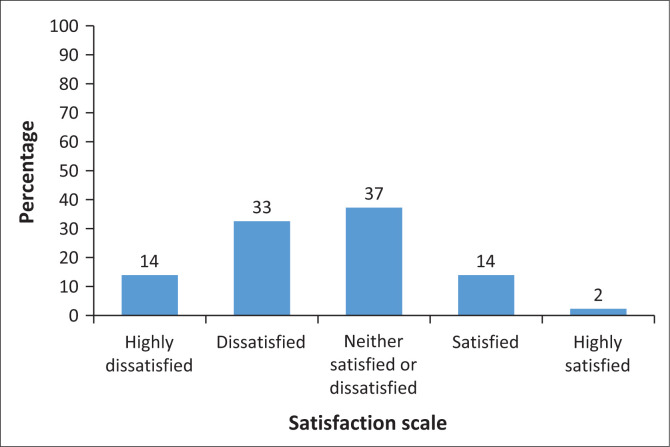
How satisfied are you with your interventional radiology training to date? (*N* = 86).

Only a minority of participants were satisfied with their IR training (16%) (responded as satisfied and highly satisfied) (Figure 5). The majority of registrars were neither satisfied nor dissatisfied (37%). Fifth-year registrars were most likely to be dissatisfied with their IR training compared to the first-year (*p* = 0.003), third-year (*p* = 0.003) and fourth-year (*p* = 0.03) registrars. There was no correlation between gender and their level of satisfaction with IR training (*p* = 0.205). In addition, when compared to the rest of the registrars, supernumerary registrars reported a lower satisfaction with their IR training (*p* < 0.001).

The participants were evenly split when answering the question on whether there should be a separate training programme for IR from diagnostic radiology (51%, Yes; 49%, No). There was also no correlation between those interested in IR (responded as highly likely and likely) and support for a separate training programme (*p* = 0.683).

**FIGURE 6:**
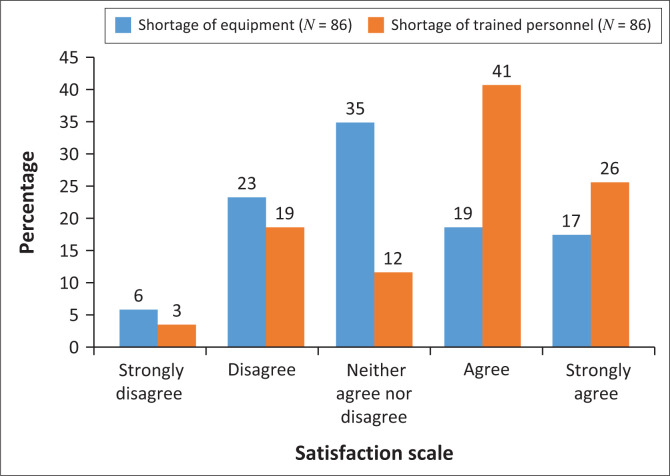
Shortage of equipment and trained personnel (*N* = 86).

A relatively large proportion of participants reported experiencing both a shortage of equipment and a lack of trained personnel, with the shortage particularly pronounced for trained personnel (Figure 6). There was however no significant correlation between shortage of equipment and the satisfaction of training (*p* = 0.065).

### Thematic analysis

The survey’s open-ended questions concerned the challenges encountered by participants and the potential for improvements in their IR training. Table 3 presents multiple emerging themes for each question.

**TABLE 3:** Thematic analysis.

Questions	Themes	Subthemes	Participants’ insights
What areas have potential for improvement in your IR training?	Inadequate IR training	Need for formalisation and structure	‘Standardised guidelines and appropriate referral guidelines’; ‘Lack of a formal training programme’; ‘Structured curriculum’; ‘Formal/structured training rotations’
		No interventional radiology	‘All areas, IR rotation does not exist in my institution’
		Mentorship and supervision	‘Mentorship and supervision’; ‘Lack of supervision’; ‘Performing procedures unsupervised’
	Practical training facilities	Inadequate facilities	‘Lack of equipment’; ‘Broken equipment needs to be replaced’
		Need for multiple training tools	‘Skills training by consultants through lectures, demonstrations and workshops’; ‘On-site skill labs, Internal case discussions’; ‘Virtual sessions and workshops’
	Exposure and hands-on experience	Lack of exposure	‘More time and hands-on teaching’; ‘Lack of exposure’
		Infrequent exposure	‘We don’t do it frequently enough, so you forget what you learned in the first rotation’,
		Wide range of procedures	‘Biopsy’; ‘Percutaneous drainage’; ‘Embolisation’; ‘Musculoskeletal intervention’; ‘oncology intervention’; ‘neuro-interventions’
What challenges do you face in IR training?	Exposure and hands-on experience	Time constraints	‘Not enough time in IR’; ‘Time to do procedures’; ‘Too much other work needs to be done’
		Lack of opportunities	‘Consultants compete with registrars for training’
	Resource constraints	Lack of resources	‘Resource constraints’; ‘Resource, equipment and staff’
		Insufficient staffing	‘Lack of seniors, lack of teaching’; ‘Lack of body interventionalists’; ‘Only one interventional consultant’; ‘Insufficiency porter and nursing support services’

IR, interventional radiology.

## Discussion

Many radiology registrars expressed interest in pursuing a career in IR. However, most reported insufficient exposure to IR and dissatisfaction with their current training. This study achieved a response rate of 45%, with a 7% margin of error, which is considered acceptable. For context, a similar study by McGuinness et al. reported a response rate of 37%.^11^ A meta-analysis on internet-based surveys found that response rates typically fall within the range of 25%–30%.^15^

The current study included an equal number of male and female participants, with no statistically significant correlations in reported interest, exposure, performance of supervised versus unsupervised procedures, or satisfaction with training. These findings are consistent with a global study across eight IR societies (UK, United States, Europe, Asia, Australia, Middle east, Canada and others), which also found no gender differences in IR training.^5^ However, despite equal training exposure, there remains a global gender disparity, with women being underrepresented in the field of IR.^4^

Globally, there are differences in the level of interest in the field of IR. Compared to a similar study in New Zealand conducted in 2006, where only 23% of registrars were interested in IR, this study found a much higher level of interest (40%).^11^ This disparity is likely multifactorial where differences in training exposure, perceived career prospects and opportunities may have played a role. Both studies cited financial considerations and exposure to the field as key factors attracting trainees to IR. McGuiness et al. also identified long hours, radiation risk and stress as deterrents.^11^ This study did not address deterrents, but found no significant correlation between interest and factors such as hours of work, procedural-based work, level of stress, patient interaction and radiation exposure. This may suggest a consistent perception of these factors as potential drawbacks across the studies.

Most participants reported limited and a few reported no exposure to IR. Two of the nine universities surveyed had no IR services, exposure or training. While registrars perform more supervised procedures over time, most still report inadequate exposure as they progress through training. The number of unsupervised angiographic procedures did not correlate with the year of study, as these registrars were likely outliers having atypical experiences. In Germany, trainees requested at least 6 months in an IR rotation during diagnostic radiology training, along with a structured curriculum, feedback, and guidance by senior interventionists.^1^ In contrast, South African registrars reported an average of 6.7 weeks in an IR rotation, with many stating that this was insufficient. Training in SA also faces significant resource and staffing constraints compounded by a lack of a structured and nationally standardised curriculum. The current training structure in SA remains insufficient, highlighting the need to improve both the quality and quantity of exposure.

Only a minority of participants were satisfied with their IR training. In contrast, a study conducted in Germany found that most participants believed that their institution provided adequate learning conditions and infrastructure for IR,^1^ with a satisfaction rate of 45%, compared to just 16% in our study. Another study covering regions such as the UK, Europe, the United States and the Middle East also reported high overall satisfaction,^5^ highlighting disparities in IR training satisfaction and exposure between SA and other regions around the world.

A systematic review assessing postgraduate medical education in sub-Saharan Africa found that satisfaction with training is low in many medical fields and not just IR.^16^ This mainly stemmed from limited resources and infrastructure for specialised training, shortage of trained faculty and inconsistent supervision, poor access to clinical training and procedural opportunities, and high workload and stress among trainees.^12^

Interventional radiology training in SA differs from other countries in many ways. South Africa offers shorter rotation durations, lacks structured curricula, has resource constraints, and limited certification pathways. As a result, mentorship-based learning is the primary training method in SA. In contrast, structured programmes such as fellowships are standard in the United States, UK, and Europe.^5^

A major challenge in SA is resource constraints and the lack of trained faculty, which likely leads to low procedural exposure and inconsistent supervision. In contrast, international programmes provide dedicated IR training centres and simulation-based learning, ensuring hands-on skill development.^17^ In addition, the shortage of IR specialists and training opportunities may contribute to high emigration rates, while countries with structured programmes offer clear career pathways.^5^ SA’s lack of board-certified IR qualification may result in trainees pursuing certification abroad. These limitations highlight key barriers to developing structured IR training programmes in SA.

The strong interest in IR among radiology registrars in SA contrasts sharply with the limited exposure and low training satisfaction, highlighting the need for structured IR training programmes. Interest in IR often correlates with exposure.^5^ McGuiness et al. emphasised that early IR exposure not only enables the trainees to start gaining important skills but also fosters mentorship, encouraging more registrars to pursue IR as a career.^11^ Introducing exposure during undergraduate level may enhance interest in the field.^15^ Overall, increasing exposure, particularly early in training, could significantly boost interest and support the growth of the IR workforce.^5^

International collaboration with Africa has played an important role in facilitating postgraduate medical education.^16^ An example is the Tanzanian IR initiative.^7^ It was initiated by first performing a baseline assessment using the IR readiness assessment tool for global health.^18^ This tool helps to assess the readiness of a facility to initiate an IR training programme. An IR fellowship programme was then developed. This programme was certified by the Tanzanian government and offers a 2-year Master of Science (MSc) in IR with three trainees per year.^7^ A completed residency in diagnostic radiology is a requirement.^7^ The training consists of in-person hands-on training with visiting international teams as well as formal didactics with regular exams.^7^

A qualitative study conducted in the United States highlighted several key components for effective IR training, many of which were also identified as areas of improvement in this study. Those components included exposure to and teaching in a broad mix of cases with procedural skills training, gradual expansion of registrar autonomy as competence develops, exposure to inter-specialty collaboration (including multidisciplinary conferences such as vascular conferences) and rotations related to IR including fibroid clinics and radiation oncology clinics, as well as access to patient management guidelines that support clinical decision-making, procedure indications and contraindications, and consideration of alternative interventions.^17^

By adapting curricula from successful international programmes, drawing selectively from African initiatives as in Tanzania, and learning from local models, facilities in SA can develop comprehensive and widely available IR training programmes. The initiative in Tanzania successfully addressed resource constraints, shortage of trained faculty as well as board certification. Meanwhile, the new fellowship in SA could offer valuable insights into strategies for maintaining sustainable programmes in SA.

These strategies should include registrars and consultants. Integrating structured and benchmarked IR training into diagnostic radiology training would better prepare prospective IR specialists, while formal IR fellowship programmes would advance the basic training and increase the number of IR specialists in SA. For those not pursuing IR as a subspeciality, they could still build a foundation for performing minor procedures, enhancing their diagnostic radiology careers.

Where local training options are limited, radiologists can pursue certification through the European Board of Interventional Radiology (EBIR).^19^ This requires 2 years of IR training, including at least 1 year after diagnostic radiology training, along with a set number of procedures and competency assessments.^17^ Registering South African training hospitals with the European Board could provide structured certification pathways.^19^

Reintroducing growth opportunities within the state sector by implementing formal IR fellowship programmes could help retain consultants not only in the public sector but also within SA, thereby reducing the potential emigration of skilled professionals. Lastly, currently there is a paucity of research in the field of IR in SA, which needs to assess a broad range of topics such as understanding IR-related conditions specific to the South African population, the number of practicing interventional radiologists in the country and their scope of practice, as well as healthcare system limitations to help tailor training programmes to address local challenges effectively. In addition, studying the accessibility and impact of IR procedures across different regions could guide resource allocation and policy development. By investing in research, SA can strengthen its IR training, improve patient outcomes, and define the role of the South African interventional radiologists.

### Limitations

The sample size, although still acceptable, may limit the generalisability of findings. A small proportion of participants did not complete certain survey questions, including 14 non-responses for questions on training satisfaction, equipment availability, and personnel shortages, and 5 non-responses for the question on IR exposure. This pattern of missing data may reflect uncertainty, limited experience, or disengagement with specific aspects of exposure and training, therefore should be considered when interpreting the findings.

Selection bias may have occurred, as not all registrars are affiliated with the RSSA, and newly appointed registrars may not yet be members. In addition, response bias may be present, as registrars interested in IR may have been more inclined to respond. Although qualitative responses were included, the depth of qualitative analysis was limited, and richer insights may have been gained through interviews or focus groups.

## Conclusion

This national study highlights a strong interest in IR among radiology registrars training in SA, yet reveals widespread dissatisfaction with current training. Key barriers include the absence of structured curricula, limited procedural exposure, resource constraints and a shortage of trained faculty. Compared to international models, South African registrars receive significantly less dedicated IR training, hindering skill development and career progression.

By addressing these key barriers, strengthening mentorship, and adapting local and international models, SA can significantly improve dedicated IR training and grow a skilled IR workforce. Coordinated action by academic institutions, professional bodies, and government stakeholders is essential to establish IR as a valuable and cost-effective pillar of the national healthcare system.
